# Reply to: “Preoperative Carbohydrate Loading on Outcomes after Cardiac Surgery: A Flawed Meta-Analysis. Comment on: The Effect of Preoperative Carbohydrate Loading on Clinical and Biochemical Outcomes after Cardiac Surgery: A Systematic Review and Meta-Analysis of Randomized Trials. *Nutrients* 2020, *12*, 3904”

**DOI:** 10.3390/nu12123905

**Published:** 2020-12-21

**Authors:** Katarzyna Kotfis, Dominika Jamioł-Milc, Karolina Skonieczna-Żydecka, Marcin Folwarski, Ewa Stachowska

**Affiliations:** 1 Department of Anesthesiology, Intensive Therapy and Acute Intoxications, Pomeranian Medical University, 70-111 Szczecin, Poland; katarzyna.kotfis@pum.edu.pl; 2 Department of Human Nutrition and Metabolomics, Pomeranian Medical University in Szczecin, 71-460 Szczecin, Poland; karzyd@pum.edu.pl (K.S.-Ż.); ewa.stachowska@pum.edu.pl (E.S.); 3 Department of Clinical Nutrition and Dietetics, Medical University of Gdansk, 80-210 Gdansk, Poland; marcinfol@gumed.edu.pl; 4 Home Enteral and Parenteral Nutrition Unit, General Surgery, Nicolaus Copernicus Hospital, 80-210 Gdansk, Poland

We appreciate the thoughts and questions posed by Drs Dileep N Lobo and Girish P Joshi [1] in relation to our manuscript entitled ‘The Effect of Preoperative Carbohydrate Loading on Clinical and Biochemical Outcomes after Cardiac Surgery: A Systematic Review and Meta-Analysis of Randomized Trials [2]. Regarding protocol and registration, we cannot agree with the conclusion that the study is non-compliant with the PRISMA statement. According to the Prisma 2009 checklist it is recommended that the authors should ‘indicate if a review protocol exists, if and where it can be accessed (e.g., Web address), and, if available, provide registration information including registration number’ [3]. This does not necessarily mean that a meta-analysis is bound to be registered, but only that the protocol is available and that the registration number should be provided if available. For our analysis, a pre-defined protocol was created in advance. The authors believe that the meta-analysis should not be excluded from publication based only on the fact of its registration in a prospective registry. We agree that it is recommended that there be a single stated primary endpoint, with the other predetermined endpoints being secondary. However, it is acceptable to use more than one primary endpoint if it is justified, or to use composite primary endpoints for clarity [4]. 

We wholeheartedly agree that the studies included in the meta-analysis were heterogenous, as is usually the case, due to differences in the methodology between studies and the different techniques used [5,6]. A study by Sokolic et al. [6] was not meta-analyzable due to the fact that the outcome measures were expressed as median and IQR. Due to the methodology of a meta-analysis, we evaluated the studies providing only means and standard deviations (in case of continuous variables). When evaluating other outcomes, i.e., the use of inotropic drugs, we indeed included the study by Lee et al. [5], who reported data on patients undergoing off-pump coronary artery bypass grafting. As when we took that factor into consideration (and excluded that study), the results we obtained were still significant (RR = 0.8, 95%CI: 0.692 to 0.924, Z value = −3.027, *p* = 0.02), therefore, we decided to include this study in the final synthesis.

It is true, as underlined by Drs Dileep N Lobo and Girish P Joshi [1], that the volume of carbohydrate loading used in different studies varied widely and ranged from 200 to 1200 mL, with variable timing of administration. We decided not to narrow the volume of carbohydrate loading, as to really reflect the clinical situation, which substantially differs between different centers. Regarding only ‘in house’ knowledge, we conducted meta-regression on that variable and found no impact of these parameters on effect sizes. The results of this meta-regression were not presented in the paper, because the number of studies was very limited. As was meta-analyzed, the volume of carbohydrate loading, the timing of administration or the type of comparator had no effect on outcomes such as exogenous insulin units (IU) in intensive care unit (ICU) and inotropic drug use, as indicated by the *I*^2^ and *p* values (with low heterogeneity for both SMD and DM or RR values that refers to the variation in study outcomes between studies). 

The authors of the correspondence letter have indicated that the preparation of carbohydrate used was not uniform, as two studies used maltodextrin in combination with omega-3 fatty acids. In both studies, Feguri et al. [7] and Feguri et al. [8] (the same clinical trial), patients were randomly assigned to a control group (water as comparator), CHO group, omega-3 group (intraoperative infusion of ω-3 PUFA) and CHO+omega-3 group. In our study however, only one arm, i.e., the CHO group and control group, were included in the synthesis. The other arms were not considered at all.

It has been underlined that the comparator in the control group varied—administration of water versus fasting. We decided not to narrow the comparator type, so as to really reflect the clinical situation, which substantially differs between study centers.

Drs Dileep N Lobo and Girish P Joshi [1] indicate that a recent meta-analysis has suggested that carbohydrate loading provides no benefits over adequate preoperative hydration [9]. Our analysis was performed only in patients undergoing cardiac surgery. Of note, we did not conduct a network meta-analysis. 

Regarding the information on perioperative care or use of ERAS pathways in the studies included, we would like to underline that none of the studies included in the meta-analysis mentioned the implementation of ERAS pathways other than carbohydrate loading. Standard surgical and anesthetic procedures were used in each study. None of the papers included in the meta-analysis described other aspects of perioperative care, e.g., the implementation of nutrition after surgery.

We agree that there were reports of the trials with the same trial registration number (NCT03017001) which had overlapping patients. The variables analyzed by Feguri et al. [8] were: incidence of POAF, infection, total duration of surgery, CPB time, CC time, acute myocardial infarction (AMI), cerebrovascular accident (CVA), hospital mortality; need for vasoactive drugs in the operating room and in the ICU; blood sugar level, amount of exogenous insulin, C-reactive protein (CRP), interleukins 6 and 10 (IL-6 and IL-10), bronchial aspiration during induction of anesthesia; bleeding in the first 12 h of recovery in the ICU; perioperative use of packed red blood cells and/or blood components; length of stay in the ICU; duration of mechanical ventilation; length of postoperative hospital stay. While in the Feguri et al. [7] study, the additional data were PONV at ICU and EVA encephalic vascular accident, none of these results were used in duplicates. The Feguri et al. and studies were not simultaneously included in any statistical analysis of statistically significant and insignificant outcomes (see Figures 2–8 and Supplementary Tables S2 and S3).

Regarding the conclusions, we decided to utilize standardized mean differences and difference in means in parallel. We put the interpretation only on the basis of differences in means (as expressed in real units, minutes and hours, respectively). We did not evaluate SMD effect sizes in raw units. As for the small sample sizes, while performing GRADE [10] evaluation, the small study group was taken into account and mentioned in the footnotes and within the limitation section.

Overall, we are aware of the limitations of our study, which is why we emphasized some aspects in Sections 4.4 and 4.5 (description of heterogeneity of CHO loading). Consequently, in our study, we do not indicate specific recommendations regarding CHO (volume, timing) in cardiac surgery, but only paid attention to the potential beneficial effects and indicated the directions of future research.
